# MPI-PHYLIP: Parallelizing Computationally Intensive Phylogenetic Analysis Routines for the Analysis of Large Protein Families

**DOI:** 10.1371/journal.pone.0013999

**Published:** 2010-11-15

**Authors:** Alexander J. Ropelewski, Hugh B. Nicholas, Ricardo R. Gonzalez Mendez

**Affiliations:** 1 Pittsburgh Supercomputing Center, Carnegie Mellon University, Pittsburgh, Pennsylvania, United States of America; 2 Department of Radiological Sciences, University of Puerto Rico School of Medicine, San Juan, Puerto Rico, United States of America; Georgia Institute of Technology, United States of America

## Abstract

**Background:**

Phylogenetic study of protein sequences provides unique and valuable insights into the molecular and genetic basis of important medical and epidemiological problems as well as insights about the origins and development of physiological features in present day organisms. Consensus phylogenies based on the bootstrap and other resampling methods play a crucial part in analyzing the robustness of the trees produced for these analyses.

**Methodology:**

Our focus was to increase the number of bootstrap replications that can be performed on large protein datasets using the maximum parsimony, distance matrix, and maximum likelihood methods. We have modified the PHYLIP package using MPI to enable large-scale phylogenetic study of protein sequences, using a statistically robust number of bootstrapped datasets, to be performed in a moderate amount of time. This paper discusses the methodology used to parallelize the PHYLIP programs and reports the performance of the parallel PHYLIP programs that are relevant to the study of protein evolution on several protein datasets.

**Conclusions:**

Calculations that currently take a few days on a state of the art desktop workstation are reduced to calculations that can be performed over lunchtime on a modern parallel computer. Of the three protein methods tested, the maximum likelihood method scales the best, followed by the distance method, and then the maximum parsimony method. However, the maximum likelihood method requires significant memory resources, which limits its application to more moderately sized protein datasets.

## Introduction

Increases in the quantity of molecular sequence data available for analysis, with over 1,000 complete genomes available at NCBI's Genome Project Resource, has brought with it both the ability and need to perform phylogenetic analyses on larger, more complex data sets. These large-scale phylogenetic studies provide unique and valuable insights into the molecular and genetic basis for important medical and epidemiological problems such as drug resistance [1], molecular receptor function [2] as well as important questions about the origins and development of physiological features in present day organisms [3], [4].

These studies use a multiple sequence alignment to represent the evolutionary history of a protein, gene, chromosome, or genome. This alignment is represented as a matrix to reconstruct a phylogenetic tree. This tree would ideally be a binary tree with unequal branch lengths. With molecular data the nodes represent speciation/mutation or duplication events, and the branch lengths the time of evolution. A recent paper [5] compared 31 orthologs across 191 species using an automated procedure to reconstruct the tree of life. The tree reconstruction used a “*supermatrix of 8090 positions for 191 species*”. Both of these tasks – constructing an optimal multiple sequence alignment and searching for the optimal phylogenetic tree – are known to be NP-complete [6].

The major methods of phylogenetic tree search are Distance Matrix based methods, Maximum Parsimony, Maximum Likelihood (ML), and Bayesian methods. The four methods are computationally intensive, particularly the ML, and Bayesian methods using Markov Chain Monte Carlo (MCMC) techniques to determine posterior probabilities (PP). Because the numbers of trees to be searched increases in a factorial manner with the number of sequences included in the multiple sequence alignment, alternate approaches that use heuristic methods to accelerate the search for the optimal tree have been explored. These heuristic searches produce reliable results, but without the certainty of arriving at the optimal solution.

In order to determine the reliability of a phylogeny and to make inferences about the evolutionary history derived from the tree the main methods are Bayesian posterior probabilities and percent support for tree nodes using resampling methods, i.e. bootstrap percentages (BP). Also bootstrapped percentage posterior probabilities (BP-PP) have been advocated as providing a better estimator than PP alone, which often yield a number of false positives [7]. Consensus phylogenies based on the bootstrap and other resampling methods are a crucial part of the new analyses that attempt to infer macroevolutionary events [8], as well as the required input for many new statistical analyses [9]. Resampling procedures use substantial computer resources and require significant turnaround time even for modest traditional datasets in molecular phylogenetics. Typically this entails an additional factor of 100–1000 or more in the running time owing to the bootstrap iterations. Today, typical BP in many large trees range from 100% to 50%, sometimes even lower. By increasing the number of bootstrap replicates, one can obtain a more reliable measure of the variance of the phylogenetic bootstrap p-value [10]. In order for the error in a binary phylogeny BP with values at 50–100% to be less than ±5% at 95% confidence, at least 400 replications must be used. For a ±2% error at least 2,000–2,500 replications are required at these BPs. For ±1% error at 90% BP approximately 3,500 replicates are needed. By increasing the number of bootstrap replicates, one can obtain a more reliable measure of the variance of the phylogenetic bootstrap p value [10]. Yet, limitations on computational resources are often cited in the literature as barriers to using the bootstrap method in large-scale phylogenetic studies [7], [11], [12]. With the rapidly expanding interest and need for these calculations, parallel versions of the software used to carry out the resampling calculations becomes a critical need.

While resampling itself requires a linear increase in the amount of computer time necessary for a particular calculation, the increased dataset size made possible by the new data collection methods causes the needed computer time to increase substantially as well. First, to find the optimal or at least a near optimal tree requires exploring or sampling the space of all possible trees which increases factorially with the number of sequences, while computing the pairwise distances between the sequences in the (resampled) data increases as the square of the number of sequences. This is by itself a significant factor since the increase in dataset size associated with the new data collection methods can be over an order of magnitude. Further, with more sequences and the factorial increase in the number of possible trees it is desirable to make a corresponding increase in the number of resampled datasets examined. Thus, the calculations under consideration can reach into weeks and even months for turnaround time on modern single processor workstations. As stated by Williams, Bader, Moret and Yan, “*depending on a user's current needs, an analysis can be short (i.e., 24 hours) or it could run for several months*” [13]. Sometimes calculations are not carried to their full extent because of the number of times a program must be run. For example, Douady, Delsuc, Boucher, Doolittle and Douzery state in their paper: “*For computing time reasons (i.e., running 2,500 times MrBayes), BP_Bay_ were only computed for the 25 data sets showing the greatest contrast between BP_ML_ and PP*” [7]. (MrBayes is a program for computing trees using Bayesian MCMC, and BP_Bay_, BP_ML_ are bootstrap percentages for posterior probabilities and maximum likelihood respectively). In more recent work Talavera and Castresana (2007) were testing the G-Blocks program for trimming alignments and how the phylogenies improved, and they wrote: “*Due to heavy computational requirements of the bootstrap analyses, the number of simulations was reduced to 150*” [14]. The only way to carry out such calculations on the number of datasets required to make robust statistical claims in a reasonable amount of time is parallelization.

Parallel and/or distributed algorithms for ML and Bayesian methods have been developed in the last few years and include DPRml [15], MultiPhyl [16], pIQPNNI [17], parallel MrBayes [18], ClustalW-MPI [19], TREE_PUZZLE [20], and RaxML [21], [22], [23], [24]. Most of these methods address the issues of computational complexity and of the tree search only. The program RaxML is one of the most developed. It provides heuristic construction of the phylogenetic tree using Maximum Parsimony. Then it goes through a maximum likelihood optimization of the tree [21], [22]. It provides very good likelihood estimates [23]. Extremely large datasets using nucleic acid sequences have been used for constructing ML phylogenetic trees using this approach. Recently the developers added a heuristic bootstrap calculation that appears to perform well in the cases used to benchmark that software [24]. A recent paper carried out the phylogenomic analysis of the cystatin superfamily [25]. They used RaxML [23] and the Neighbor-Joining algorithm from MEGA4 [26]. They found that “*the NJ method with uncorrected distances was found to produce better resolution of evolutionary relationships in the cystatin superfamily than the more complex ML method*” [25]. Russo, Takezaki and Nei [27] showed that the efficiencies of the NJ, ME, MP and ML methods in obtaining the correct tree were nearly the same when amino acid sequence data were used. The most important factor in constructing reliable phylogenetic trees appeared to be the number of amino acids or nucleotides used. As a final consideration it should be noted that although the ML methods appear to be more accurate in reconstructing a phylogeny, there may exist several trees for a given set of aligned sequences that maximize the likelihood of the phylogeny [28].

Our group is interested in the evolutionary history of proteins and how to integrate this history with sequence and structural information to understand function and mechanisms of enzymes and other proteins. For example we have studied the aldehyde dehydrogenase family evolution, structure and function with computationally intensive methods [29], [30], [31]. For our specific purposes, proteins are the more appropriate molecules to use for the study of phylogenies [32]. Furthermore, Russo, Takezaki and Nei [27] showed that when sequences diverge using the protein sequence yields more accurate reconstructions of the true phylogeny. Because the protein families that we study typically have dozens or hundreds of members, we have the specific need to carry out phylogenetic studies on protein families of these sizes. Furthermore, in our studies we desire the ability to compare trees from a variety of different methods using a common bootstrapped dataset. These factors led us to parallelize the resampling calculations using the protein-oriented routines of the PHYLIP phylogenetic suite.

The PHYLIP package is one of the most comprehensive sets of tools freely available for use in phylogenetic studies [33]. According to the release notes of the package, PHYLIP has been distributed since 1980, and has over 20,000 registered users. The PHYLIP package can be used with protein (and nucleic acid) sequences and includes distance matrix methods, parsimony methods, and maximum-likelihood methods to search for phylogenetic trees using both heuristic and exact algorithms. PHYLIP also includes routines that allow for a variety of resampling techniques that include bootstrapping, jackknifing, and permutation of characters. It also allows for consensus trees to be reconstructed from the resampled data analyses by use of strict consensus or several variants of majority rule consensus. Since bootstrapping of datasets in the PHYLIP suite is a separate process, the same bootstrapped dataset can be used as input into different phylogenetic methods, enabling the comparison of trees created by different methods from a common bootstrapped framework.

This paper reports our initial parallelization of the computationally intensive bootstrap calculations discussed above on datasets of various sizes using the PHYLIP suite of phylogenetic software. Our parallel code enables the phylogenetic study using a statistically robust amount of bootstrap replicates for large protein families using the distance and parsimony methods as well using a statistically robust amount of bootstrap replicates for moderate sized protein families using the maximum likelihood method implemented in PHYLIP.

## Results

The test datasets (containing 1,000 bootstrap replicates generated with the PHYLIP seqboot program) used to illustrate the performance of the parallel codes are: 1) a 375 residue, single-gene alignment of cytrochrome B from the mitochondrial genome from thirteen species of plasmodium (*1/13*); 2) a 375 residue, single-gene alignment of cytrochrome B from the mitochondrial genome from twenty five species of plasmodium (*1/25*); 3) a 1139 residue, three-gene alignment consisting of the cytrochome B, cytochrome oxydase 1, and cytochrome oxydase 3 genes from the mitochondrial genome from twenty five species of plasmodium (*3/25*); 4) a 389 residue single gene alignment of 60 ABCG transporters from a variety of species (*1/60*); and 5) a 356 residue, single gene alignment from a set of 121 g-protein alpha inhibitory subunits from fungi (*1/121*). These bootstrapped datasets are available as supplemental files. In addition to these test datasets, the Tree-of-Life dataset of Ciccarelli, Doerks, von Mering, Creevey, Snel and Bork (2006) was used as an example of a large post-genomic dataset. The dataset includes 191 species and is 8089 columns long.

Code performance for the parallel-bootstrap PHYLIP protpars, protdist, and proml programs (called MPIprotpars, MPIprotdist, and MPIproml), run with the default program parameters on the test datasets is summarized in Tables 1 and 2. The timings shown in the tables were run on the SGI Altix 4700 shared-memory NUMA systems at the Pittsburgh Supercomputing Center. The PSC ALTIX systems are configured with blades holding two Itanium 2 Montvale 9130M dual core processors. Each core had a clock rate of 1.66GHz and the four cores on each blade shared 8 Gigabytes of local memory. Due to the constraints and limitations of these systems dataset (*1/60*) was only run once with the maximum likelihood method and datasets (*1/121*) and the Tree-Of-Life dataset were not run using the maximum likelihood method.

**Table 1:** Benchmark Results for Parallel PHYLIP programs.

Program	Genes	Sequences	Alignment Length	Max Memory (Gb)	Elapsed Time				
					4	8	16	32	64
MPIproml	3	25	1139	16	143130	72456	35863	18938	9968
	1	25	375	7	130452	66116	34580	17285	9645
	1	13	375	2.5	20004	10342	5335	3025	1618
MPIprotdist	1	121	356	<0.1	42416	21233	11806	5507	2941
	1	60	389	<0.1	11239	5626	2852	1485	818
	3	25	1139	<0.1	5542	2786	1419	757	460
	1	25	375	<0.1	1884	947	484	259	174
	1	13	375	<0.1	493	250	130	75	57
MPIprotpars	1	121	356	<0.1	33518	17105	8815	5115	3040
	1	60	389	<0.1	9645	5033	2610	1479	843
	3	25	1139	<0.1	1418	760	408	262	229
	1	25	375	<0.1	451	256	157	118	119
	1	13	375	<0.1	89	50	33	28	34

Program is the parallel version PHLIP program. Genes refers to the number of genes present in the alignment. Sequences refers to the number of sequences present in the alignment. Alignment Length refers to the length of the underlying multiple sequence alignment being analyzed. Max Memory is the maximum memory consumed by the parallel program listed as a per-processor value in gigabytes. Elapsed Time is the elapsed time in seconds for the parallel code to run on the Altix system for the number of cores shown.

**Table 2:** Benchmark Results for Tree-Of-Life Dataset.

Program	Sequences	Alignment Length	Elapsed Time	
			64	128
MPIprotdist	191	8089	109394	62606
MPIprotpars	191	8089	209762	123284

Program is the parallel version PHLIP program. Sequences refers to the number of sequences present in the alignment. Alignment Length refers to the length of the underlying multiple sequence alignment being analyzed. Elapsed Time is the elapsed time in seconds for the parallel code to run on the Altix system for the number of cores shown.

### Memory Requirements

The protein parsimony and distance calculations require a minimal amount of memory, even for the larger calculations. All of these test cases required less than 100 Megabytes of memory. On the other hand, the maximum likelihood calculations all required a substantial amount of memory. The test cases required approximately 2.5Gb (1/13), 7Gb (1/25), and 16Gb (3/25) of memory per core on the Altix systems. It is important to note that while the Altix NUMA architecture permits global memory sharing, it is accomplished (through decreased efficiency) at the expense of idle cores. To perform maximum likelihood calculations efficiently would require hardware configured with substantially more memory per processor than the configuration cited in this paper. In addition a full exploration of the memory management schema used by the maximum likelihood code is needed to further understand this issue and evaluate if improvements such as those undertaken by the fastdnaml code [34] may be possible in the proml code.

### Scaling

**Figure 1:**
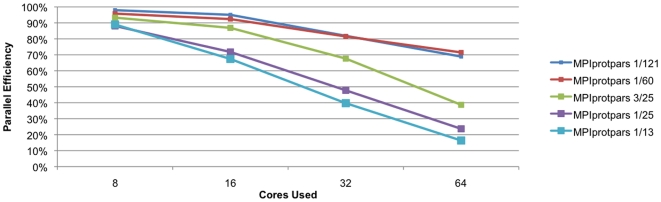
Scaling of MPIprotpars. Scaling of the parallel implementation of the PHYLIP parsimony code (MPIprotpars) on the five test datasets discussed in this paper. The vertical axis indicates the percentage efficiency compared to the four core run while the horizontal axis indicates the number of processors used.

**Figure 2:**
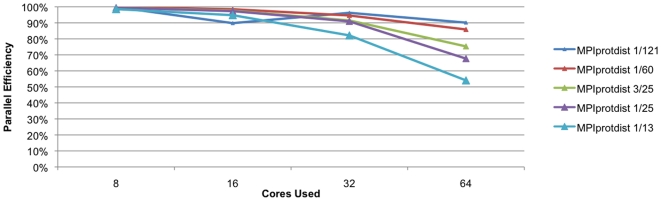
Scaling of MPIprotdist. Scaling of the parallel implementation of the PHYLIP distance code (MPIprotdist) on the five test datasets discussed in this paper. The vertical axis indicates the percentage efficiency compared to the four core run while the horizontal axis indicates the number of processors used.

**Figure 3:**
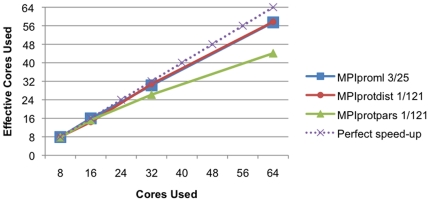
Speed-up of parallel bootstrapped phylogeny calculations. Speedup of the parallel implementation of the PHYLIP codes (MPIprotdist, MPIprotpars and MPIproml) on the largest dataset used for each method discussed in this paper. The vertical axis indicates the effective number of processors compared to the four-processor run while the horizontal axis indicates the number of processors used.

The figures in this paper show the efficiency and effective speedup of the parallel implementation on the test datasets. Both efficiency and effective speedup in the figures are defined in terms of the four processor run. The efficiency at *n* processors is defined as:
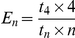
where *n* is the number of processors utilized and *t* is the wallclock time of the run. The effective speedup (in processors) at *n* processors is defined as:
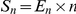
The CPU intensity of the algorithm and the size of the dataset both have a rather obvious effect on the scaling of the code. *Figure 1* shows the efficiency of the parallel implementation of the parsimony code, while *Figure 2* shows the efficiency of the distance code. Of these two methods, the distance method is more efficient and scales better than the parsimony method on the test datasets. This is not surprising given that the distance method is in general more CPU intensive than parsimony on protein family datasets. Table 1 shows in detail the results obtained for the datasets tested except for the the Tree-of-Life dataset. For small datasets with only a few dozen species, the parsimony code scales well only to about 8–16 processors while the distance code on these same small datasets scales well to about 32–64 processors. For larger datasets, which would require more computation, both of these methods will scale to substantially more processors. With the larger datasets (1/60 and 1/121) the parsimony codes scales only to 70% efficiency using 64 processors, while the distance code scales to approximately 90% efficiency. This means that we are effectively using 44 processors for the parsimony calculations and 58 processors for the distance calculations (*Figure 3*). Improvements in the parsimony algorithm would be required to improve efficiencies. It is important to note that calculations that take a few days on these datasets using a state-of-the-art desktop workstation are reduced in time to a calculation that can be performed over lunchtime in a state-of-the-art cluster. Table 2 shows the results for the Tree-of-Life dataset. We can see that both the distance and parimony codes scale only to 128 processors with this dataset. The Tree-of-Life dataset, the largest used in this paper, required less than two days of computational time for 1,000 bootstrap replicates using 128 processors.

**Figure 4:**
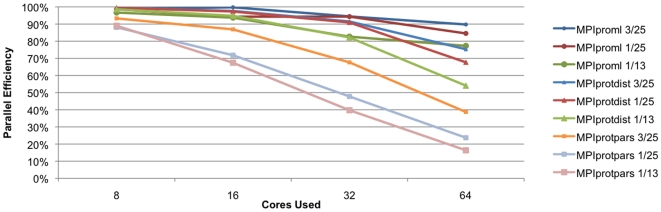
Scaling of MPIproml. Scaling of the parallel implementation of the PHYLIP maximum likelihood code (MPIproml) compared with the parallel distance (MPIprotdist) and parallel parsimony (MPIprotpars) methods. The vertical axis indicates the percentage efficiency compared to the four core run while the horizontal axis indicates the number of processors used.

*Figure 4* shows the scaling of the maximum likelihood method on test cases (1/13), (1/25), and (3/25) compared with the scaling of the same test cases using the distance and parsimony methods. A single maximum likelihood calculation on the (1/60) dataset using 32 processors for computing and the memory from 480 processors on the Altix system took approximately 25.5 hours and 30 Gb of memory per compute processor (data not shown). Due to the excessive resources required for the calculation, we did not explore the largest dataset with the likelihood approach. Again, the more CPU intensive method (likelihood) scales better than either the distance or the parsimony codes given the same bootstrapped dataset. But it should be noted that the distance code approaches the efficiency of the maximum likelihood approach with the large datasets that are barely accessible by the latter method due to the time required because of the physical memory configuration of the system. While resource limitations, particularly memory preclude us from examining the scaling of the likelihood method with larger datasets further in this paper, we are confident that the likelihood method will continue to scale well beyond the number of processors shown in this paper when larger datasets are used. However, simply the ability to perform likelihood calculations on moderately sized protein datasets in a reasonable amount of time is very beneficial in fulfilling the needs of research groups such as ours that is interested in studying protein phylogenies.

## Methods

Our focus in this project was to use parallelization to effectively increase the number of bootstrapping replications that could be performed on large protein datasets. Specifically, we wanted to create a parallel implementation that:

- Required minimal changes to the original PHYLIP source code, but still allowed a substantial speedup in the processing of large bootstrapped datasets.
- Made use of the MPI message-passing library which would enable the parallel implementation to work on a wide variety of computer architectures.
- Used a methodology could be transferred to other computationally intensive routines in the PHYLIP Package, such as the routines in the package that are relevant to the study of evolution at the nucleotide level.

How much code to modify is always an issue when porting an existing widely used serial code to a parallel platform. From a code maintenance standpoint, having the parallel and serial versions of the code maintained from a common source will help to ensure that the latest enhancements in the serial code are also available in the parallel code. On the other hand, to gain maximum parallel efficiency codes often need to be extensively re-written, often in a way that forces the development of special parallel versions that are not fully compatible with the serial code. We wanted to avoid this situation as the PHYLIP package is actively being developed and enhanced, and we had a strong desire to minimize any future incompatibilities that the parallel code would have with the serial code.

Also driving the parallelization methodology decision were the coding practices used in the PHYLIP package. From a programming perspective, one of the nice features of the PHYLIP package is its consistency in programming style across the package's 30+ component programs that make up the PHYLIP suite. This coding style makes it easy to apply knowledge learned from examining the source code that makes up one program in the package into another. The use of multiple input and output files and the extensive use of global variables in the package also contributed to the selection of the parallel approach.

The PHYLIP package sets program parameters and options by reading from the standard input and by reading in data sets from an input file and optionally categories and weights from separate files. Data read from standard input and the input files are read into numerous global variables that are referenced by multiple functions in the package. It is the extensive use of these global variables that hinders parallelizing the code without an extensive rewrite. However, the manner in which bootstrapping is implemented in the PHYLIP package makes an alternative parallel approach possible without an extensive recoding. Conceptually, inside each of the PHYLIP programs that makes use of bootstrapped datasets is a loop that looks similar to the following:

For each bootstrap dataset:

1. Read the bootstrap dataset from input file into global variables
2. Perform the tree reconstruction calculation
3. Write the results to output file

Thus, the code is essentially using global variables as scratch space: loading each dataset into this global space, using this global space to store intermediate results and then writing out the final results from this global space to the output file. Each independent tree-reconstruction bootstrap calculation is performed under the same parameters. In order to parallelize the code, one only needs to ensure that each processor receives the same input parameters, works on unique datasets, and write the results in a coordinated fashion to a common output file. This can be easily implemented if each of the parallel processors being utilized has access to a file system.

To keep the code consistent with the serial version, we developed data primitives in MPI to distribute parameters and input file data to the processors. These data primitives are contained in an auxiliary file used in the parallel implementation called MPICopyfile.c. There are two minor restrictions placed on the parallel code that is not in the original PHYLIP codes. Those restrictions are: 1) that the default input and output filenames that the PHYLIP package expects (*infile*, *outfile*, *outtree*, etc.) must be used and 2) that the program input must be placed in a file named *stdin*. These minimal restrictions are necessary because of the way that these files are distributed to the processors. While the program is running, each node accesses its own input file and writes the data to its own output file. Prior to termination the output files are collected into a single output file. The general parallelism used in this implementation is as follows:

1. The lead processor distributes the stdin file to all processors
2. The lead processor distributes the input file to all processors
3. Each processor advances the input file pointer to a chunk of unique bootstrapped data based on the MPI processor number and the total number of bootstrapped datasets.
4. Each processor evaluates its unique bootstrapped data:
  - The unique dataset is read from the local input file into global variables
  - The parsimony, distance, or maximum likelihood calculation is performed
  - The results are written to a local output file
5. The lead processor collects the results from all the processors and writes the data into single output file. The lead processor collects any additional output files produced by the method in the same manner.

This general parallelism is implemented exclusively in the files MPIprotdist.c, MPIprotpars.c and MPIproml.c, which are modifications to the original protdist.c, protpars.c, and proml.c, files as well as the auxiliary file containing the data primitives (MPIcopyfile.c). No other source codes in the PHYLIP package were modified to create the parallel implementations.

### How to install and run the software

The coding approach described above enables the parallel PHYLIP code to be run on virtually any UNIX computer in which the MPI library is available and has been installed. A Makefile and instructions are included in the distribution to compile and link the software on platforms where the standard MPI compiler wrappers (ie. mpicc) have been installed as well as the Altix system. To install the distribution, first download the compressed tar file (MPIsrc.tar.gz) from the web site (File S1). Next uncompress the file using the gunzip command (“gunzip MPIsrc.tar.gz”) and unpack it with the tar command (“tar xvf MPIsrc.tar”).

To compile and link the software on platforms where the standard MPI compiler wrappers (ie. mpicc) have been installed, change your working directory to the MPIsrc/src subdirectory (via “cd MPIsrc/src”). Next, copy the Makefile.mpicc file to a file called Makefile (“cp Makefile.mpicc Makefile”). Then use the make command to build compile and link the code with the appropriate MPI library (“make all”). In general we have found that only minor modifications to the compile and link lines in the Makefile are required to run the code on parallel platforms that do not use the standard MPI wrappers (including the Altix platform used to run the test cases.) To compile on the Altix platform simply copy the Makefile.Altix file to a file called Makefile (“cp Makefile.Altix Makefile”). Then use the make command to build, compile and link the code with the appropriate MPI library (“make all”).

In the test directory of the distribution are several input files that can be used to test the installation. The files ending in “.boot” are bootstrapped input files, the files ending in “.stdin” are the program input files and the files ending in “.job” are UNIX (PBS) script files that can be used to run the parallel versions of the programs.

The bootstrapped datasets used for the performance measures described in this paper are located in the “paper” subdirectory of the distribution (Files S2, S3, S4, S5 and S6).

To make full use of the parallelized routines described in this paper, the serial PHYLIP suite is also recommended to be installed to be able to generate bootstrapped datasets and to analyze the output files produced by the parallel programs.

#### Availability

Users can download a tar file containing the parallelized PHYLIP routines described in this paper from the website www.nrbsc.org/downloads/. The serial version of the PHYLIP suite is available from Joseph Felsenstein's website: http://evolution.genetics.washington.edu/phylip/

#### Future code optimizations

The current parallel implementation is reliant on each processor having access to a file system because each processor needs to read and write its own unique data. In general, it is the file input and output that limits the scaling of the code. With adequate per-processor memory available, the possibility of using internal (memory) files to store the input data might be an obtainable optimization that may improve the scaling of the codes on moderate sized datasets. In lieu of using internal files, the potential exists to internalize the physical creation of the bootstrap dataset instead of relying on the external seqboot program. Internalizing the bootstrap creation would simultaneously reduce the amount of message passing data that needs to be distributed as well as greatly reducing the volume of data that needs to be read by the programs.

The output files produced by each of the programs vary substantially and typically are much larger than the input files. The case for using internal files for these datasets is less clear. For example, the size of the largest output file from the (1/121) distance runs described in this paper is 149Mb, (or about three times as large as the largest input file) and the largest parsimony output file is over 5.8 Gb or about 100 times as large as the largest input file. While compression algorithms may be used to reduce the physical size needed to store this output data, compression is accomplished at the cost of increased CPU time. Even with compression, the amount of memory required to store these output files internally may be prohibitively large enough to render this potential optimization infeasible for output datasets on datasets of the size that we are interested in studying.

In addition, a full exploration of the memory management schema used by the maximum likelihood code is needed to further understand the amount of memory used by the code. Any improvement that can be made in this area will increase the size of datasets that can be examined by the method as memory is the principal limitation of the code that we encountered in our studies. We know that improving the memory management of the original PHYLIP dnaml code is one of the major optimizations of the fastdnaml code [34], thus if these optimization techniques are also relevant to the protein maximum likelihood code, substantial improvements in memory usage and program performance may be possible. However, the proml code differs substantially in many key ways from the fastdnaml code most notably being the hidden markov model features described in Felsenstein and Churchill [35]. Thus, while it may be unclear if the fastdnaml memory improvements are possible, the techniques are at least something to explore.

#### Future research directions

We have shown that parallel computing can enable the use of very large number of bootstrap replicates for the study of moderate sized protein phylogenetic datasets when using the robust maximum likelihood method in relatively short time frames (typically less than 48 hours). Large protein datasets are similarly addressed using the parsimony and distance methods. We have also illustrated how a valuable legacy code in the biosciences can be effectively parallelized with minimal code changes. Future work will include explorations on the maximum likelihood code's memory management schema, code optimizations including more efficient file handling and the exploration of the supertree approach to maximum likelihood [12]. Furthermore, although the MPI version of the codes for analyzing DNA sequences have been implemented and are available, they should be thoroughly characterized and optimized in future work.

In the post-genome era the availability of vast amounts of data from genomes, transcriptomes, proteomes, and metabolic networks has opened new avenues to study life and evolution. A detailed understanding of the phylogenies implicated by the genes, proteins, networks, and organisms is crucial to this endeavor. The majority of the phylogenetic methods being applied are focused on the nucleic acid composition of genes, genomes, and transcriptomes, thus will have difficulties to account for constraints on the gene products in the physical realm such as protein structure, biophysics, and function issues among others. In general the signal-to-noise-ratio of methods based on protein characteristics will be better than methods like the 16S-RNA-phylogenies. This effect is due to the stable conservation of protein structure and architecture when compared to the coding sequence. An additional advantage is that longer evolutionary time-scales will become accessible for study. As noted by Yang, Doolittle and Bourne [36] proteins such as metabolic enzymes, cytoskeleton-proteins, or histones can be expected to evolve even more slowly making longer time scales amenable to evolutionary studies. As expressed by Mayr [37], evolution during selection acts upon advantageous changes of the phenotype and not necessarily upon changes in the genotype. The phenotype is, however, to a large extent determined by the encoded proteome. Protein-based approaches are more concerned with this advantage. Furthermore, they appear to produce more reliable phylogenies than those based on nucleic acids [27].

We have been studying large protein superfamilies with several hundred members [30]. The recent work on the cystatin superfamily found over 2100 members of this protein across all the kingdoms [25]. These large protein datasets can only be studied in a reasonable amount of time using the methods described here. For Example, we have carried out the distance calculation for the Tree-of-Life dataset [5], which includes 31 protein families from 191 species, in 17.4 hours using the MPI-Protdist code with 1,000 bootstrap replicates. Carrying out 1,000 bootstrap replicates serially on datasets this large can take months of computational time, and re-running the phylogenies when one adds a new subfamily or additional sequences can become an extreme burden. With the new parallel methods, phylogenies on datasets such as these can be computed over a few days.

The exploration of phylogenies with new information theoretic approaches such as the Jensen-Shannon divergence [38] to look for functionally important residues and sites, key residues that define protein subfamilies (Nicholas, unpublished results), as a distance measure for protein domain phylogenies [39], or the combinatorial entropy measure to group protein alignments into subfamilies and map the defining residues to structures [40] has become an important complement to phylogenetic studies that requires studying large protein families to be able to understand the evolution of structure and function within them. The software presented in this paper will significantly improve our capability to carry out this kind of research.

## Supporting Information

File S1 MPI Source Code for the Parallel PHYLIP methods (in .tar.gz format).
(1.11 MB GZ)

File S2 Test dataset (containing 1,000 bootstrap replicates) from a 356 residue, single gene alignment from a set of 121 g-protein alpha inhibitory subunits from fungi (1/121).
(6.47 MB GZ)

File S3 Test dataset (containing 1,000 bootstrap replicates) from a 389 residue single gene alignment of 60 ABCG transporters from a variety of species.
(6.51 MB CDX)

File S4 Test dataset (containing 1,000 bootstrap replicates) from a 375 residue, single-gene alignment of cytrochrome B from the mitochondrial genome from thirteen species of plasmodium.
(0.70 MB GZ)

File S5 Test dataset (containing 1,000 bootstrap replicates) from a 375 residue, single-gene alignment of cytrochrome B from the mitochondrial genome from twenty five species of plasmodium.
(1.02 MB GZ)

File S6 Test dataset (containing 1,000 bootstrap replicates) from a 1139 residue, three-gene alignment consisting of the cytrochome B, cytochrome oxydase 1, and cytochrome oxydase 3 genes from the mitochondrial genome from twenty five species of plasmodium.
(3.14 MB GZ)
